# Explorando los desafíos: causas de rechazo de potenciales donantes de órganos en Colombia

**DOI:** 10.7705/biomedica.7730

**Published:** 2026-03-02

**Authors:** Andrea Gómez-Montero, Andrea García-López, William Cruz-Mususú, Luisa Vargas, Ximena Escobar, Milena Orellano-Salas, Santiago Cabas, Fernando Girón-Luque

**Affiliations:** 1 Departamento de Investigación, Colombiana de Trasplantes, Bogotá, D. C., Colombia Departamento de Investigación Colombiana de Trasplantes Bogotá D. C Colombia; 2 Fundación Donar Colombia, Bogotá, D. C., Colombia Fundación Donar Colombia Bogotá D. C Colombia; 3 Departamento de Cirugía de Trasplantes, Colombiana de Trasplantes, Bogotá, D. C., Colombia Departamento de Cirugía de Trasplantes Colombiana de Trasplantes Bogotá D. C Colombia

**Keywords:** obtención de tejidos y órganos, trasplante de órganos, muerte encefálica, factores de riesgo, selección de donante, consentimiento informado, Organ and tissue procurement, organ transplantation, brain death, risk factors, donor selection, informed consent

## Abstract

**Introducción.:**

El trasplante de órganos es vital para mejorar la calidad de vida de los pacientes con insuficiencia orgánica, pero la escasez de donantes es un desafío global. En Colombia, la demanda supera la oferta, lo cual aumenta la mortalidad de aquellos pacientes en las listas de espera.

**Objetivo.:**

Analizar las causas de rechazo de potenciales donantes de órganos, según la evaluación de Fundonar en tres regionales del país durante el año 2022.

**Materiales y métodos.:**

Se llevó a cabo un estudio transversal descriptivo basado en datos retrospectivos de posibles donantes reportados a la Red de Donación y Trasplantes de Colombia. Se incluyeron 1451 casos evaluados. Las causas para rechazar un donante potencial se agruparon en tres categorías: médicas, médico-legales y logísticas. Se utilizó estadística descriptiva, se evaluó la normalidad de las variables y se compararon los donantes con contraindicaciones en cada regional mediante el análisis de varianza. Se utilizó el *software* R, versión 4.0.3.

**Resultados.:**

De los 1451 donantes evaluados, 849 tenían causas para el rechazo. De estos, el 29,8 % presentó falla multiorgánica, el 20,8 % tenía alguna comorbilidad y el 15,3 % no cumplía con los criterios diagnósticos de muerte encefálica.

**Conclusión.:**

La mayoría de las causas médicas de rechazo podrían reconsiderarse sopesando los riesgos y los beneficios para los receptores. Un enfoque personalizado y multidisciplinario, con criterios basados en la evidencia y decisiones en tiempo real adaptadas a Colombia, podría mejorar las tasas de aprobación de donación de órganos.

## Abstract (en)

**Introduction.:**

Organ transplantation is crucial for improving the quality of life of patients with organ failure, but the shortage of donors is a global challenge. In Colombia, the demand exceeds the supply, increasing mortality rates on waiting lists.

**Objective.:**

To evaluate the causes of contraindications in potential organ donors assessed by *Fundonar* across three regional centers in the country during 2022.

**Materials and methods.:**

A descriptive cross-sectional study was conducted based on retrospective data from potential donors reported to the *Red de Donación y Trasplantes de Colombia.* A total of 1451 cases were evaluated. The causes of contraindications were categorized into medical, medico-legal, and logistical categories. Descriptive statistics were used, the normality of the variables was assessed, and contraindicated donors from each regional center were compared using ANOVA. R software, version 4.0.3, was used.

**Results.:**

Of the 1451 donors evaluated, 849 had contraindications. Among these, 29.8% presented multiple organ failure, 20.8% had a comorbidity, and 15.3% did not meet the diagnostic criteria for brain death.

**Conclusion.:**

Most of the medical contraindications could be reconsidered by balancing risks and benefits for the recipients. A personalized and multidisciplinary approach, with evidence-based criteria and real-time decisions tailored to Colombia, could improve organ donation approval rates.

El trasplante de órganos es una estrategia costo-efectiva que mejora la calidad de vida de los pacientes con una enfermedad terminal ^1^^-^^5^. A nivel mundial, se practican anualmente casi 150 000 trasplantes de órganos, que representan solamente el 10 % de la demanda en las listas de espera ^6^^,^^7^. Esta discrepancia entre oferta y demanda aumenta los tiempos de espera y, en consecuencia, aumenta la morbimortalidad ^8^^,^^9^. Por este motivo, muchos criterios que antes excluían a un donante se han vuelto más flexibles en las últimas décadas ^10^^-^^13^.

Durante el 2022, en Colombia se reportaron 3663 pacientes en la lista de espera para recibir donación de algún órgano, de los cuales 1190 (32,5 %) recibieron un trasplante. Pese a esto, se presentaron 110 fallecimientos de las listas de espera durante ese año ^14^, lo que resalta la necesidad de optimizar los procesos de donación y de establecer estrategias para mitigar dicha mortalidad.

La Fundación Donar Colombia (Fundonar Colombia) es una organización para la obtención de órganos con presencia en tres áreas del país, llamadas coordinaciones regionales de trasplantes (en adelante, "regionales"). Estas regionales, identificadas como la 1, 2 y 5 (figura 1), se encargan de evaluar los pacientes reportados en la Red de Donación y Trasplantes de Colombia. Juntas, contribuyen con cerca del 70 % de la actividad de donación en el país ^15^.

**Figura 1:**
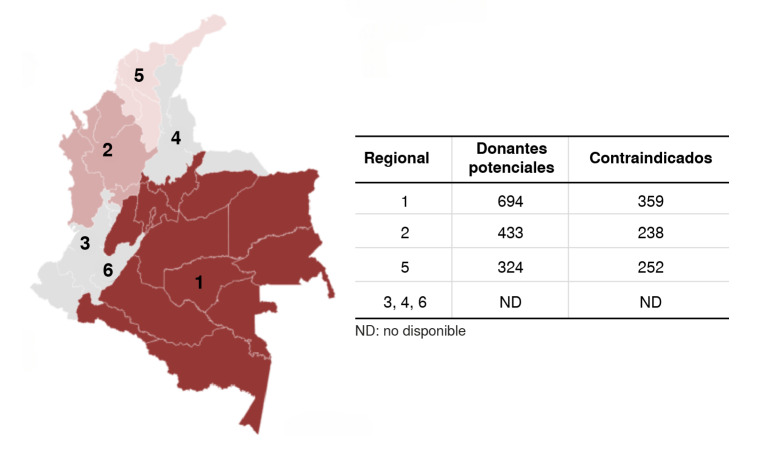
Número de donantes potenciales y donantes rechazados en las diferentes regionales de Colombia en el año 2022

La información que se muestra en el mapa refleja los datos a los que Fundonar tiene acceso. Otras regionales también cuentan con información relevante, aunque actualmente no tenemos acceso a sus datos.

En este estudio se informan las principales causas del rechazo de potenciales donantes de órganos y su frecuencia, según la evaluación por Fundonar Colombia en dichas regionales durante el 2022.

## Materiales y métodos

### Diseño y población

Se trata de un estudio descriptivo de corte transversal de potenciales donantes de órganos, evaluados por Fundonar Colombia entre el 1° de enero y el 31 de diciembre de 2022, y reportados por las tres regionales del país (regionales 1, 2 y 5). A lo largo del texto, se utiliza la expresión "potenciales donantes" de forma indistinta para referirse a los posibles y potenciales donantes según la nomenclatura establecida por la Organización Mundial de la Salud (OMS) en la ruta crítica de la donación ^16^.

### Recolección de datos

Se hizo una recolección retrospectiva de las características sociodemográficas a partir de:

1. los registros de atención de los potenciales donantes reportados a la red de trasplantes y evaluados por los médicos de Fundonar Colombia,
2. los diagnósticos clínicos principales,
3. la obtención del consentimiento para la donación y
4. las causas de rechazo.

Las causas para rechazar un donante potencial se agruparon en tres categorías: médicas, médico-legales y logísticas. Cada categoría incluye varias causas específicas y un paciente podía presentar más de una razón de rechazo en una o más categorías. Para los análisis, se consideró solamente la causa principal de rechazo.

### Análisis estadístico

Se hizo un análisis descriptivo para informar las variables de interés. La distribución normal se evaluó usando la prueba de Kolmogorov-Smirnov. Los datos no paramétricos se expresaron mediante la mediana y el rango intercuartílico. Los grupos de potenciales donantes rechazados en las tres regionales se compararon utilizando la prueba de análisis de la varianza. Los análisis se llevaron a cabo con el *software* estadístico R, versión 4.0.3.

### Consideraciones éticas

Por considerarse un estudio con riesgo mínimo según la Resolución 8430 de 1993 ^17^, el estudio fue eximido de la exigencia de consentimiento informado por el Comité de Ética Dexa Diab (aprobación N°01485). Los datos analizados fueron anonimizados, con lo cual se garantizó la confidencialidad y el manejo ético de los datos.

## Resultados

### Características de los donantes potenciales

En el 2022, Fundonar Colombia evaluó 1451 donantes potenciales de 29 municipios de las tres regionales. Predominó el sexo masculino (n = 899, 62,0 %) y el grupo etario de los 41 a los 65 años (n = 633, 43,6 %), seguido por el grupo de los 19 a los 40 años (n = 466, 30,7 %). El 83,6 % (n = 1213) de los donantes provenía de las áreas urbanas y el 87,8 % (n = 1274) se encontraba hospitalizado en la unidad de cuidados intensivos.

Los diagnósticos neurológicos más frecuentes fueron el accidente cerebrovascular hemorrágico (n = 599, 41,3 %) y el trauma craneoencefálico (n = 451, 31,1 %). En el cuadro 1 se presentan las características principales del total de los pacientes evaluados. Además, en la figura 1 se presenta la distribución de los donantes de las regiones de Colombia, la cual evidencia una concentración en las grandes ciudades del país.

**Cuadro 1:** Caracterización del grupo de donantes potenciales evaluados por Fundonar Colombia en el año 2022

		n (%)
Sexo		
	Masculino	899 (62,0)
	Femenino	552 (38,0)
Grupo de edad (años)		
	0-5	34 (2,3)
	6-12	24 (1,7)
	13-18	59 (4,1)
	19-40	446 (30,7)
	41-65	633 (43,6)
	>65	255 (17,6)
Tipo de ubicación		
	Urbana	1213 (83,6)
	Rural	238 (16,4)
Contexto clínico		
	Urgencias	177 (12,2)
	UCI	1274 (87,8)
Diagnóstico neurológico		
	ACV hemorrágico	599 (41,3)
	TCE	451 (31,1)
	ACV isquémico	138 (9,5)
	Hipoxia	128 (8,8)
	Tumor cerebral	71 (4,9)
	Desconocido	14 (1,0)
	Otro	50 (3,4)

UCI: unidad de cuidados intensivos; ACV: accidente cerebrovascular; TCE: trauma craneoencefálico

### Exclusión del protocolo

**Figura 2:**
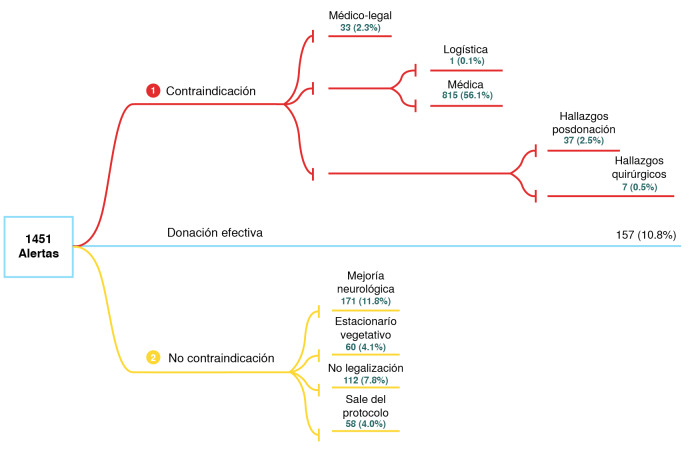
Diagrama de flujo de alertas de donación por contraindicaciones, donación efectiva y sin contraindicación, en tres regionales de Colombia en el año 2022

Del total de donantes potenciales evaluados, 231 se excluyeron del protocolo de evaluación: 60 por un estado neurológico estacionario -es decir, estado de coma, vegetativo, mínima conciencia, enclaustramiento- y 171 por mejoría de su estado neurológico. En 58 casos, otro equipo de trasplantes se encargó del manejo del paciente. También, se excluyeron 112 pacientes con diagnóstico de muerte, según los criterios neurológicos, por no cumplir con los requisitos para la donación. En la figura 2 se presenta la distribución de las 1451 alertas analizadas.

### Características de los donantes rechazados

Un total de 849 (58,05 %) pacientes se consideraron no aptos para ser potenciales donantes, en su mayoría hombres (n = 510, 60,1 %), con una distribución predominante en el grupo etario de los 41 a los 65 años (n = 339, 39,9 %). El 84,6 % (n = 718) provenía de las áreas urbanas y el 87,8 % (n = 745) se encontraba hospitalizado en la unidad de cuidados intensivos. Las principales causas de deterioro neurológico fueron el accidente cerebrovascular hemorrágico (n = 343, 40,4 %) y el trauma craneoencefálico (n = 205, 24,1 %). Se encontraron diferencias estadísticamente significativas en todas las variables analizadas al comparar las tres regionales, excepto en la causa de deterioro neurológico (cuadro 2).

**Cuadro 2:** Caracterización del grupo de potenciales donantes rechazados evaluados por Fundonar Colombia en tres regionales de Colombia en el año 2022

		Rechazados			
		Regional 1	Regional 2	Regional 5	Total
		n (%)	n (%)	n (%)	n (%)
Total		359 (42,3)	238 (28,0)	252 (29,7)	849 (100)
Sexo*					
	Masculino	206 (24,3)	158 (18,6)	146 (17,2)	510 (60,1)
	Femenino	153 (18,0)	80 (9,4)	106 (12,5)	339 (39,9)
Grupo de edad (años)*					
	0-5	13 (1,5)	8 (0,9)	9 (1,1)	30 (3,5)
	6-12	8 (0,9)	5 (0,6)	5 (0,6)	18 (2,1)
	13-18	11 (1,3)	9 (1,1)	10 (1,2)	30 (3,5)
	19-40	88 (10,4)	66 (7,8)	74 (8,7)	228 (26,9)
	41-65	141 (16,6)	94 (11,1)	104 (12,2)	339 (39,9)
	>65	98 (11,5)	56 (6,6)	50 (5,9)	204 (24,0)
Tipo de ubicación*					
	Urbana	325 (38,3)	206 (24,3)	187 (22,0)	718 (84,6)
	Rural	34 (4,0)	32 (3,8)	65 (7,7)	131 (15,4)
Contexto clínico*					
	Urgencias	21 (2,5)	73 (8,6)	10 (1,2)	104 (12,2)
	UCI	338 (39,8)	165 (19,4)	242 (28,5)	745 (87,8)
Diagnóstico neurológico					
	ACV hemorrágico	153 (18,0)	80 (9,4)	110 (13,0)	343 (40,4)
	TCE	75 (8,8)	81 (9,5)	49 (5,8)	205 (24,1)
	ACV isquémico	40 (4,7)	18 (2,1)	25 (2,9)	83 (9,8)
	Hipoxia	40 (4,7)	16 (1,9)	38 (4,5)	94 (11,1)
	Tumor cerebral	27 (3,2)	20 (2,4)	21 (2,5)	68 (8,0)
	Desconocido	4 (0,5)	2 (0,2)	8 (0,9)	14 (1,6)
	Otro	20 (2,4)	21 (2,5)	1 (0,1)	42 (4,9)

* Diferencias estadísticamente significativas entre los grupos UCI: unidad de cuidados intensivos; ACV: accidente cerebrovascular; TCE: trauma craneoencefálico

### Causas del rechazo

**Figura 3:**
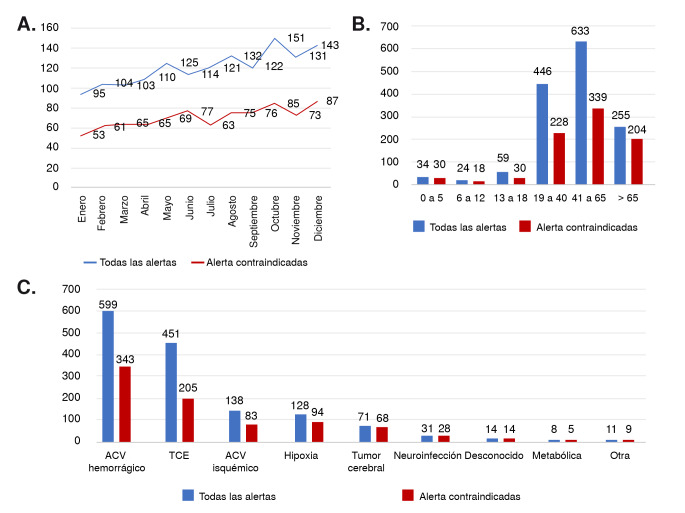
A) Alertas y contraindicaciones durante el año 2022; B) distribución por grupos etarios de las alertas y contraindicaciones; C) causas del deterioro neurológico en las alertas y contraindicaciones.

Las causas para rechazar un donante potencial se agruparon en tres categorías: médica (815 casos, 56,1 %), médico-legal (33 casos, 2,3 %) y logística (1 caso, 0,1 %). En el cuadro 3 se detallan las causas de rechazo según la categoría y la regional correspondiente. La figura 3a muestra la evolución del número de alertas y del número de donantes rechazados durante el 2022. En la figura 3b se observa una mayor frecuencia de rechazos en los grupos de adultos jóvenes, de mediana edad y adultos mayores. Sin embargo, al analizar la proporción de contraindicaciones en cada grupo etario, se observa una concentración en las edades extremas: 88,2 % (n = 30) en menores de 5 años, 75 % (n = 18) en escolares y 80 % (de 6 a 12 años) en adultos mayores.

**Cuadro 3:** Causas para rechazar un potencial donante de órganos, según la evaluación por Fundonar en tres regionales de Colombia en el año 2022

Categoría	Causa	Regional 1	Regional 2	Regional 5	Total	
		n	n	n	n	(%)
Médicas	Falla multiorgánica, inestabilidad hemodinámica	157	33	63	253	29,8
	Comorbilidades, antecedentes médicos	37	77	63	177	20,8
	Incapacidad de completar el diagnóstico	36	29	65	130	15,3
	Neoplasia confirmada o sospechada	54	32	30	116	13,6
	Sepsis, infección o sospecha de infección	31	33	12	76	8,9
	Edad	10	10	5	25	2,9
	SARS-CoV-2 confirmado	5	2		7	0,8
	Causa no clara de muerte	4	1	4	9	1,1
	HIV		4	1	5	0,6
	Hemodilución	2	1		3	0,3
	Imposibilidad de abordaje familiar	2		1	3	0,3
	Sospecha o riesgo de infección por SARS-CoV-2		2	1	3	0,3
	Conductas de riesgo	2	1		3	0,3
	Solicitud de necropsia clínica	2	1		3	0,3
	Indigencia	1	1		2	0,2
Médico-legales	Estado migratorio irregular	7	3	2	12	1,41
	Persona en custodia del Estado	3	3		6	0,71
	Maltrato infantil	3	2		5	0,59
	Alteración de la evidencia médico-legal	2		2	4	0,47
	Feminicidio	1		1	2	0,24
	Persona sin identificación	1	1		2	0,24
	Violencia sexual		1		1	0,12
	Ausencia de tutores legales en paciente pediátrico			1	1	0,12
Logísticas	Incapacidad para la gestión		1		1	0,12

ACV: accidente cerebrovascular; TCE: trauma craneoencefálico

Por otro lado, en la figura 3c se muestran los diagnósticos relacionados con deterioro neurológico en cada caso. En el cuadro 4, se detallan las causas de rechazo de los pacientes con diagnóstico confirmado de muerte encefálica, durante la evaluación del equipo de donación.

**Cuadro 4:** Causas de rechazo en pacientes con diagnóstico de muerte encefálica evaluados en Fundonar Colombia en tres regionales durante el año 2022

Muerte encefálica		Regional 1	Regional 2	Regional 5	Total	
		n	n	n	n	(%)
Médica		66	25	21	112	47,1
	Causa no clara de muerte			1	1	0,4
	Comorbilidades, antecedentes médicos	3	5	4	12	5,0
	Conductas de riesgo	1	1		2	0,8
	SARS-CoV-2 confirmado	1	1		2	0,8
	Edad	2			2	0,8
	Falla multiorgánica o inestabilidad hemodinámica	31	5	7	43	18,1
	Imposibilidad de abordaje familiar	1		1	2	0,8
	Incapacidad de completar el diagnóstico	3	3	1	7	2,9
	Indigencia		1		1	0,4
	Neoplasia confirmada o sospechada	17	4	2	23	9,7
	Sepsis, infección o sospecha de infección	7	3	4	14	5,9
	Sospecha o riesgo de infección por SARS-CoV-2		2		2	0,8
	HIV			1	1	0,4
Médico-legal		4	1	1	6	2,5
	Alteración de la evidencia médico-legal	1			1	0,4
	Ausencia de tutores legales en paciente pediátrico			1	1	0,4
	Estado migratorio irregular	1			1	0,4
	Maltrato infantil	1			1	0,4
	Persona en custodia del Estado	1	1		2	0,8
Logística			1		1	0,4
	Incapacidad para la gestión		1		1	0,4

HIV: *Human Immunodeficiency Virus*

### Contraindicaciones médicas

Las contraindicaciones médicas fueron las más prevalentes (n = 815, 56,1 %), destacándose la falla multiorgánica y la inestabilidad hemodinámica (n = 253, 29,8 %), seguidas de comorbilidades y antecedentes médicos que pueden afectar la calidad de los órganos donados (n = 117, 20,8 %). La incapacidad de completar el diagnóstico de muerte con criterios neurológicos, esencial para poder donar, en ausencia de programas de donación en muerte por criterios circulatorios en el país, representó el 15,3 % (n = 130). Algunas causas relativas, como la infección o la sospecha de SARS-CoV-2, la hemodilución o las conductas de riesgo, representaron un bajo porcentaje. Entre las neoplasias sospechosas o confirmadas, no se incluyeron los tumores del sistema nervioso central de bajo grado, como los meningiomas de grado I o II, el carcinoma basocelular de piel o el de cuello uterino, ya que estas neoplasias no generan contraindicación.

### Contraindicaciones médico-legales y logísticas

Entre las causas médico-legales (n = 33, 2,3 %), las más comunes fueron: el estado migratorio irregular del potencial donante en el país (1,41 %; n = 12), ser persona en custodia del Estado (fuerzas armadas en cumplimiento de servicio o personas con restricción de la libertad) (0,71 %; n = 6) y el maltrato infantil (0,59 % n = 5). Actualmente, además del estado migratorio irregular, en la legislación no existen contraindicaciones médico-legales absolutas, por lo que estos casos se someten a consulta con peritos del Instituto Nacional de Medicina Legal y Ciencias Forenses para recibir recomendaciones sobre su manejo. El único caso de contraindicación logística incluyó la incapacidad para la gestión (0,12 %), haciendo referencia a que no se pudo garantizar el traslado de los componentes o del personal en condiciones óptimas para llevar a cabo el proceso.

## Discusión

Este es el primer estudio en el que se describen las razones para rechazar los donantes en las primeras fases de la ruta crítica de la donación de órganos en Colombia.

Las principales causas -falla multiorgánica e inestabilidad hemodinámica, presencia de comorbilidades y falta de oportunidad en el diagnóstico- dan cuenta del 65,9 % (n = 560) de los rechazos de potenciales donantes y pueden ser intervenidas. Por otro parte, es escaso el número de causas que no se pueden intervenir, como la presencia de neoplasias, la alteración de evidencia médico-legal o el estado migratorio irregular del donante, así como algunas relativas, como la infección por COVID, la sepsis o las conductas de riesgo.

Desde inicios del milenio, la OMS ha hecho un llamado a los países para alcanzar la autosuficiencia en términos de donación y trasplante, con el fin de reducir el riesgo de prácticas como el turismo para trasplantes y el tráfico de órganos ^16^. Una de las estrategias para lograr este propósito es el concepto de la "ruta crítica de la donación", que es importante porque describe los puntos críticos de la atención en los que se descartan donantes potenciales.

En el grupo de pacientes que se reportan a la Red de Trasplantes, en el caso colombiano, cerca del 10 % llegan a ser donantes efectivos ^18^. Por tanto, el entender las causas del rechazo de donantes y el poder identificar en qué punto de la ruta se presentan, se vuelve esencial para lograr aumentar las tasas de donación y, consecuentemente, la oportunidad de hacer un trasplante.

Cerca del 30 % de las contraindicaciones se deben a inestabilidad hemodinámica, falla multiorgánica o ambas, lo que genera la necesidad de analizar el estado en el que se reportan los pacientes a la Red y el manejo del paciente neurocrítico con pronóstico incierto. Este mal estado hemodinámico y de función orgánica del paciente neurocrítico al ser reportado implica la pérdida potencial de uno de cada tres donantes reportados a la Red de Trasplantes. Aunque el país ha implementado medidas legislativas para mejorar el manejo de los potenciales donantes, no se dispone de datos previos para evaluar su impacto en esta causa de contraindicación.

Por otro lado, en el presente estudio, el 84,6 % de los casos provenía de las zonas urbanas (figura 1), lo que podría reflejar una mayor capacidad diagnóstica, así como un acceso más oportuno a las unidades de cuidados intensivos y a los equipos especializados de donación. Sin embargo, esta distribución también sugiere una posible subrepresentación de los donantes de las áreas rurales, donde las limitaciones en infraestructura hospitalaria, las barreras geográficas o los retrasos en la remisión podrían influir negativamente en la identificación y la evaluación de potenciales donantes.

Además, el 20,8 % de las contraindicaciones se debe a la imposibilidad de diagnosticar la muerte encefálica, lo cual está relacionado con las limitaciones mencionadas.

Según la experiencia de los autores, en algunos casos no se logra hacer el diagnóstico debido a hipernatremia persistente, inestabilidad del donante o falta de conocimiento de los médicos tratantes sobre el uso de herramientas diagnósticas como la ecografía Doppler transcraneal o la angiografía cerebral. En un estudio de Braksick *et al.,* se demostró que existe una amplia divergencia en el conocimiento y las prácticas de diagnóstico de muerte encefálica ^19^.

Globalmente, con criterios menos exigentes, se están aceptando más donantes, lo cual aumenta la disponibilidad de potenciales donantes y la oportunidad del trasplante para las personas en la lista de espera ^20^. Incluso, se ha demostrado que una de las estrategias más utilizadas es fortalecer las prácticas de donación de órganos en los casos de muerte circulatoria, es decir, con interrupción permanente de la circulación sanguínea y la función cardiaca y, así, reducir la pérdida de oportunidades de donación ^21^.

Por otro lado, la literatura sobre las contraindicaciones para la donación es limitada, aunque se ha incrementado recientemente. En los primeros estudios, se identificaron las limitaciones consideradas como contraindicaciones absolutas debido al riesgo de generar complicaciones para el receptor ^22^^,^^23^. Sin embargo, se ha reevaluado este riesgo y algunos pacientes con estas condiciones podrían beneficiarse del trasplante con un manejo adecuado, lo cual justifica la donación de órganos en ciertos casos ^24^. Por ejemplo, actualmente, los donantes potenciales con hepatitis C pueden donar a receptores con antecedentes de esta enfermedad ^24^^,^^25^. En el periodo analizado, no se descartaron donantes por este antecedente.

Los donantes mayores de 65 o, incluso, de 70 años, pueden tener resultados favorables si se seleccionan adecuadamente ^26^^,^^27^; sin embargo, muchos de ellos se descartan por presentar antecedentes de enfermedades crónicas no transmisibles. En este sentido, se recomienda incluir dichos donantes bajo la supervisión de equipos especializados ^20^^,^^26^.

Actualmente, existe una controversia sobre los potenciales donantes con contraindicaciones relativas, como la obesidad, la diabetes y las enfermedades cardiovasculares ^28^, en especial, cuando se trata de instituciones con recursos limitados en los que la toma de decisiones sobre la asignación de órganos es compleja ^29^. La falta de consenso en los criterios subraya la necesidad de directrices más uniformes y basadas en la evidencia ^30^^,^^31^.

En donantes potenciales con múltiples comorbilidades, el equipo médico debe evaluar de manera cuidadosa el balance entre los riesgos y los beneficios de la donación, considerando tanto la condición médica del receptor como su calidad de vida, soporte social y potencial cumplimiento del protocolo postrasplante ^6^^,^^31^. La consideración de estos elementos no solo optimiza la selección de candidatos adecuados, sino que justifica la donación de órganos como intervención terapéutica al demostrar su potencial para mejorar significativamente la supervivencia y el bienestar global del paciente.

Además, los factores de riesgo que podrían contraindicar la donación de órganos raramente se presentan de forma aislada. Por ejemplo, en el presente estudio se encontró que el 52,3 % (444 pacientes) presentaba múltiples contraindicaciones, tanto médicas como logísticas o médico-legales.

Aunque cada condición por sí sola podría no ser una contraindicación absoluta, la combinación de varias sí puede aumentar el riesgo de morbilidad y mortalidad posoperatorias ^32^. Por lo tanto, es crucial evaluar el riesgo global, considerando que estas condiciones interrelacionadas pueden afectar el resultado del trasplante ^6^^,^^26^. El uso de modelos personalizados de predicción del riesgo, que integren múltiples variables clínicas, podría mejorar la toma de decisiones y ayudar a identificar los pacientes que podrían beneficiarse del trasplante a pesar de presentar múltiples factores de riesgo ^31^^,^^33^.

En cuanto al contexto clínico, el manejo óptimo de potenciales donantes en la unidad de cuidados intensivos es fundamental para preservar los órganos y reducir la tasa de contraindicaciones por falla multiorgánica (la causa más frecuente en el 29,8 % de los pacientes). El mantenimiento de la estabilidad hemodinámica, el control estricto de las infecciones y la preservación de la función orgánica requieren un enfoque muy especializado ^34^. Por ello, es imperativo fortalecer la capacitación del personal clínico en el manejo integral del donante, garantizando la implementación de protocolos basados en la mejor evidencia disponible ^35^^,^^36^. La adecuada formación del personal de salud resulta esencial para disminuir los rechazos y maximizar la viabilidad de los órganos, lo cual contribuye al éxito de los programas de trasplantes ^37^.

En el presente estudio, se presentó un 20,8 % de rechazos por comorbilidades. Teniendo esto en cuenta, la observancia de las guías clínicas para el manejo de los donantes en la unidad de cuidados intensivos es crucial para optimizar la viabilidad de los órganos y los resultados de los programas de trasplantes ^38^. Aunque algunos donantes no puedan ser recuperados debido a daños irreversibles, en ciertos casos, como en la sepsis, un manejo adecuado puede revertir la condición crítica ^39^. Se ha demostrado que un esquema antibiótico prolongado, ajustado a los agentes patógenos involucrados, puede estabilizar los donantes sépticos y preservar la funcionalidad de sus órganos ^40^^,^^41^. Esto pone en evidencia la importancia de aplicar las guías con flexibilidad, adaptando el tratamiento a las circunstancias clínicas para maximizar el éxito del trasplante.

Las infecciones por el HIV, las hepatitis B y C, y la presencia del virus SARS-CoV-2, que anteriormente se consideraban contraindicaciones absolutas para la donación de órganos, ya no lo son debido a los avances en los tratamientos antivirales y las estrategias del manejo postrasplante. En la actualidad, la aplicación de protocolos estrictos permite la evaluación y la selección cuidadosa de donantes con estas condiciones, mitigando los riesgos y optimizando la seguridad para los receptores ^10^^-^^13^. Esta evolución en la práctica ha aumentado el número de donantes disponibles, lo cual ha mejorado significativamente las oportunidades de trasplante sin comprometer los resultados clínicos.

La decisión de proceder con un trasplante en tales circunstancias requiere un enfoque multidisciplinario y un análisis cuidadoso de los riesgos y de los beneficios ^6^^,^^26^. La medicina de trasplantes está evolucionando y las innovaciones en el análisis de los datos en tiempo real optimizan la toma de decisiones ^21^^,^^24^^,^^31^. De hecho, en el presente estudio se implementó la aplicación de auditorías de donantes, como lo recomienda la evidencia ^21^, lo que genera un referente para mejorar el proceso de selección de donantes cadavéricos en Colombia.

Se debe evaluar de forma continua cómo el Sistema de la Red Nacional de Donación y Trasplante de Órganos hace el seguimiento de los pacientes neurocríticos reportados que eventualmente podrían ser donantes ^42^^,^^43^, para que sean vigilados antes de que presenten un deterioro que los contraindique como donantes ^26^. Asimismo, se recomienda la aplicación de inteligencia artificial y de medicina de predicción en los trasplantes ^31^, y revisar y flexibilizar los criterios de rechazo, especialmente en los casos de edad avanzada y comorbilidades, para ampliar el grupo de donantes y mejorar las oportunidades de trasplante.

Es fundamental adoptar un enfoque personalizado y multidisciplinario en la evaluación de los riesgos, considerando la interacción de múltiples factores médicos. Además, es crucial desarrollar criterios uniformes, basados en la evidencia, que incorporen los avances en la toma de decisiones y que estén fundamentados en los datos en tiempo real, enfocados específicamente en el caso colombiano.

Este estudio tiene limitaciones, como el sesgo debido a la recolección retrospectiva de los datos. Además, el manejo de grandes cantidades de información podría ocasionar la ausencia de ciertos datos o errores de medición. Para abordar este problema, los investigadores revisaron minuciosamente la información y normalizaron las variables para garantizar la calidad de los datos.

Entre las fortalezas de este estudio se resaltan: 1) el tamaño de la población, que incluyó información de las regionales que llevan a cabo la mayoría de los trasplantes de órganos (70 %) en el país ^15^, por lo que se considera una cohorte representativa a nivel nacional y 2) el proceso de recolección de la información, que los investigadores estandarizaron desde un principio, lo que permitió obtener datos de mejor calidad.

Este es el primer estudio en el que se describen las contraindicaciones de los donantes para rechazarlos, en todas las fases de la vía crítica de la donación de órganos en Colombia. La mayoría de los rechazos se deben a contraindicaciones médicas, lo que se podría flexibilizar a la luz de la evidencia clínica sobre la seguridad de aceptar potenciales donantes con criterios menos exigentes, considerando los riesgos y beneficios para los receptores.
